# Efficacy of Omentum-Preserving Gastrectomy for Patients With Gastric Cancer: A Systematic Review and Meta-Analysis

**DOI:** 10.3389/fonc.2021.710814

**Published:** 2021-09-03

**Authors:** Zonglin Li, Min Song, Yejiang Zhou, Huaiwu Jiang, Linxia Xu, Zhengchuan Hu, Yi Liu, Yifan Jiang, Xin Li

**Affiliations:** ^1^Department of Gastrointestinal Surgery, The Affiliated Hospital of Southwest Medical University, Luzhou, China; ^2^Department of Laboratory Medicine, The Affiliated Hospital of Southwest Medical University, Luzhou, China; ^3^Department of Gastrointestinal Surgery, Sichuan Mianyang 404 Hospital, Mianyang, China

**Keywords:** gastric cancer, omentum-preserving gastrectomy, omentectomy, surgical outcomes, oncological outcomes

## Abstract

**Background:**

Complete omentectomy is considered to be essential in the radical gastrectomy for gastric cancer (GC), but its clinical benefit remains unclear. This study aims to evaluate the efficacy of omentum-preserving gastrectomy (OPG) for patients with GC.

**Methods:**

Studies comparing the surgical and oncological outcomes of OPG and gastrectomy with complete omentectomy (GCO) for GC up to March 2021 were systematically searched from PubMed, Web of Science, Embase, and Cochrane Library. A pooled analysis was performed for the available data regarding the baseline features, surgical and oncological outcomes. The RevMan 5.3 software was used to perform the statistical analysis. Quality evaluation and publication bias were also conducted.

**Results:**

Nine studies with a total of 3335 patients (1372 in the OPG group and 1963 in the GCO group) undergoing gastrectomy were included. In the pooled analysis, the baseline data in two groups were all comparable (p > 0.05). However, the OPG group was associated with shorter operative time (MD = −18.67, 95% CI = −31.42 to −5.91, P = 0.004) and less intraoperative blood loss (MD = −38.09, 95% CI = −53.78 to −22.41, P < 0.00001) than the GCO group. However, the number of dissected lymph nodes (MD = 2.16, 95% CI = −0.61 to 4.93, P = 0.13), postoperative complications (OR = 0.92, 95% CI = 0.74 to 1.15, p = 0.47), overall recurrence rate (OR = 0.83, 95% CI = 0.66 to 1.06, p = 0.14), peritoneal recurrence rate (OR = 0.91, 95% CI = 0.65 to 1.29, p = 0.60), 3-year relapse-free survival (RFS) rate (OR = 1.40, 95% CI = 0.86 to 2.27, p = 0.18), and 5-year RFS rate (OR = 1.21, 95% CI = 0.95 to 1.55, p = 0.12) of the two groups were comparable.

**Conclusions:**

OPG might be an oncologically safe procedure with better surgical outcomes for patients with GC than GCO. However, high-quality randomized controlled trials are needed to confirm this benefit.

## Introduction

In 2018, a large-scale randomized controlled trial (JCOG1001) reported that omentobursectomy does not provide a survival advantage over non-bursectomy (omentectomy) for patients with gastric cancer (GC) (1). Thereafter, bursectomy is not recommended for GC surgery in the guidelines of the Japanese Gastric Cancer Association (JGCA). According to the current guidelines of the JGCA (5th edition), gastrectomy with complete omentectomy (GCO) and D2 lymphadenectomy are the mainstream procedures for GC surgery (2). Nevertheless, the clinical benefit of GCO for GC remains unclear.

The greater omentum is a double sheet and has the largest peritoneal fold. Given that peritoneal dissemination is the most common recurrent type after curative gastrectomy for GC, the greater omentum is usually completely resected to eliminate microscopic cancer seeds. However, the complete removal of the peritoneum from the abdominal cavity is theoretically impossible and operationally impractical. Thus, the effect of GCO on the prevention of peritoneal recurrence may be limited. In recent years, some retrospective studies reported omentum-preserving gastrectomy (OPG), in which the greater omentum is dissected 3 cm far from the gastroepiploic arcade, whereas the greater omentum on the side of the transverse colon is preserved. These studies pointed out that GCO increases operative time, intraoperative blood loss, or postoperative complications but provides no oncological advantage over OPG for patients with GC (3–5). Therefore, further research is needed to assess the efficacy of OPG in GC surgery, but no large-scale randomized controlled trial is available to date.

This meta-analysis aims to evaluate the efficacy of OPG on the basis of the current published studies.

## Methods

This meta-analysis was carried out in line with the preferred reporting items for systematic reviews and meta-analysis (PRISMA) statement.

### Search Strategy

Studies comparing the surgical and oncological outcomes of OPG and GCO for GC up to March 2021 were systematically searched from PubMed, Web of Science, Embase, and Cochrane Library. The keywords used for the search were “gastric cancer” and “omentectomy”. Thus, the following search string was used across the above databases: “stomach neoplasms” [MeSH Terms] or “stomach” [All Fields] and “neoplasms” [All Fields] or “stomach neoplasms” [All Fields] or “gastric” [All Fields] and “cancer” [All Fields] or “gastric cancer” [All Fields] and omentectomy [MeSH Terms] or omentectomy [All Fields]. No date or language restriction was imposed. Full articles from reviews were also checked for potential articles. The search was last performed on March 17, 2021.

### Study Selection and Data Extraction

The included studies met the following criteria: (1) comparative studies about the surgical and oncological outcomes of OPG and GCO for GC surgery and (2) original research published in English. The exclusion criteria were as follows: (1) studies published as reviews, letters, case reports, animal studies, meeting abstract, surgical technique, and protocols of randomized controlled trial; (2) studies with incomplete or inaccurate data for analysis; (3) articles with a mixed study population, which led to unavailable analysis for patients with GC.

Two reviewers (ZL and MS) carried out the screening and extraction processes independently. First, studies were screened by titles and abstracts. Then, the full texts of the potential studies were checked. For eligible articles, the following information from each article was recorded: first author, publication year, country, study design, study interval, study object, sample size, and operation method. Furthermore, the following clinicopathological parameters were extracted from these studies: sex, age, body mass index (BMI), American Society of Anesthesiologists (ASA) score, pathological stage, histologic type, resected type, adjuvant chemotherapy, operation time, intraoperative blood loss, number of retrieved lymph nodes, postoperative complications, overall recurrence rate, peritoneal recurrence rate, 3-year relapse-free survival (RFS) rate, and 5-year RFS rate. Results were checked by a third author (YZ).

### Risk of Bias Assessment

The qualities of the selected studies were assessed in accordance with the Cochrane Handbook. Biases, including selection, performance, detection, attrition, reporting, and others, were evaluated. Outcomes were summarized using a bias graph.

### Statistical Analysis

The mean difference (MD) and odds ratio (OR) with 95% confidence interval (CI) were used to evaluate continuous and dichotomous variables, respectively. For studies that only reported median and range, data were converted into mean and standard deviation (SD) (6). Heterogeneity among studies was assessed using *χ* ^2^ and *I* ^2^ statistics. *I* ^2^ > 50% indicated significant heterogeneity, and the random-effects model was used. Otherwise, the fixed-effects model was performed. Funnel plots were conducted to assess publication bias. A p value < 0.05 was considered significant. All statistical analyses were performed using the RevMan 5.3 software (Cochrane, London, UK).

## Results

### Characteristics of Studies

**Figure 1:**
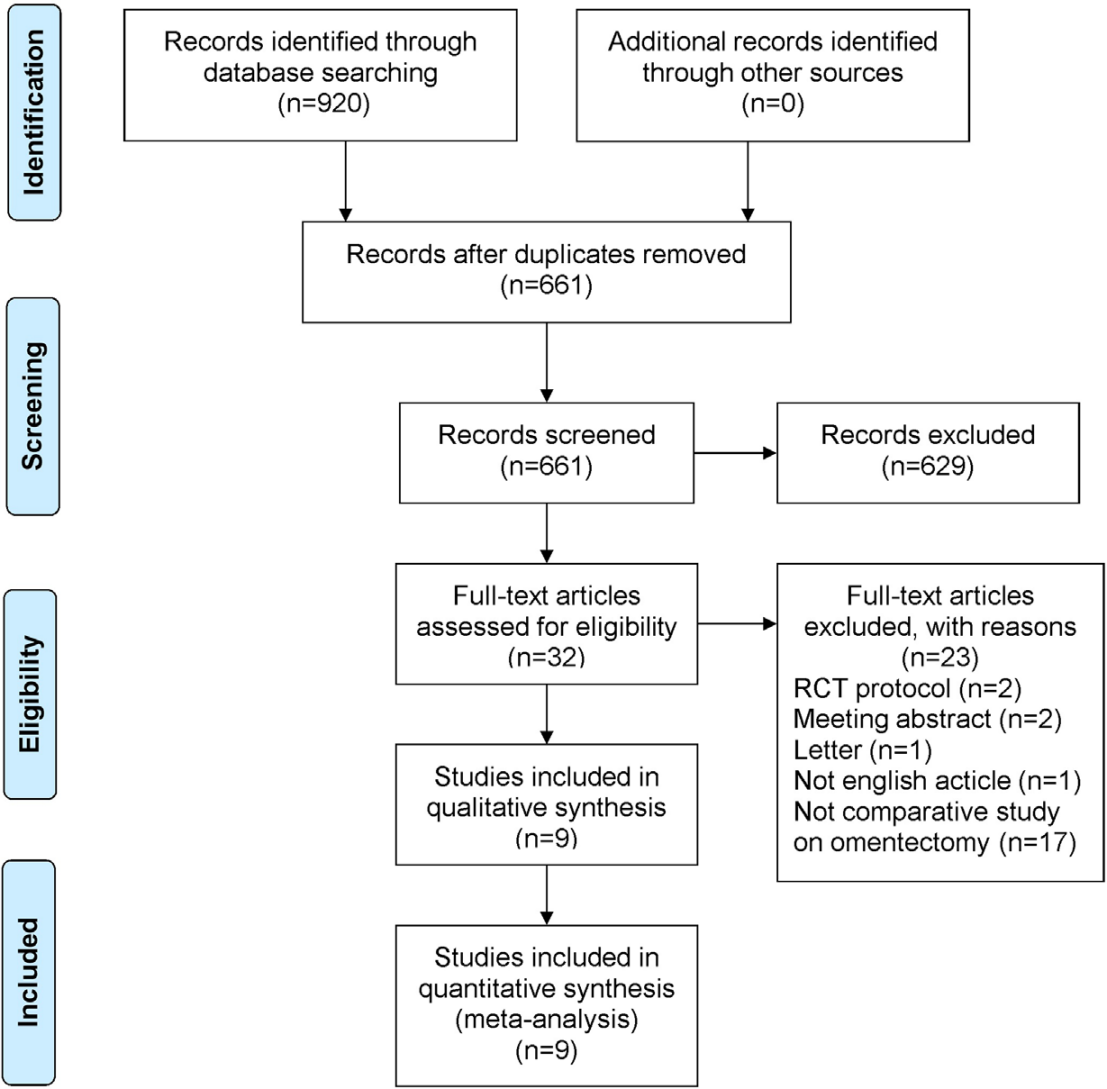
PRISMA flowchart of literature search and selection process. PRISMA, preferred reporting items for systematic review and meta-analysis; RCT, randomized controlled trial.

**Figure 2:**
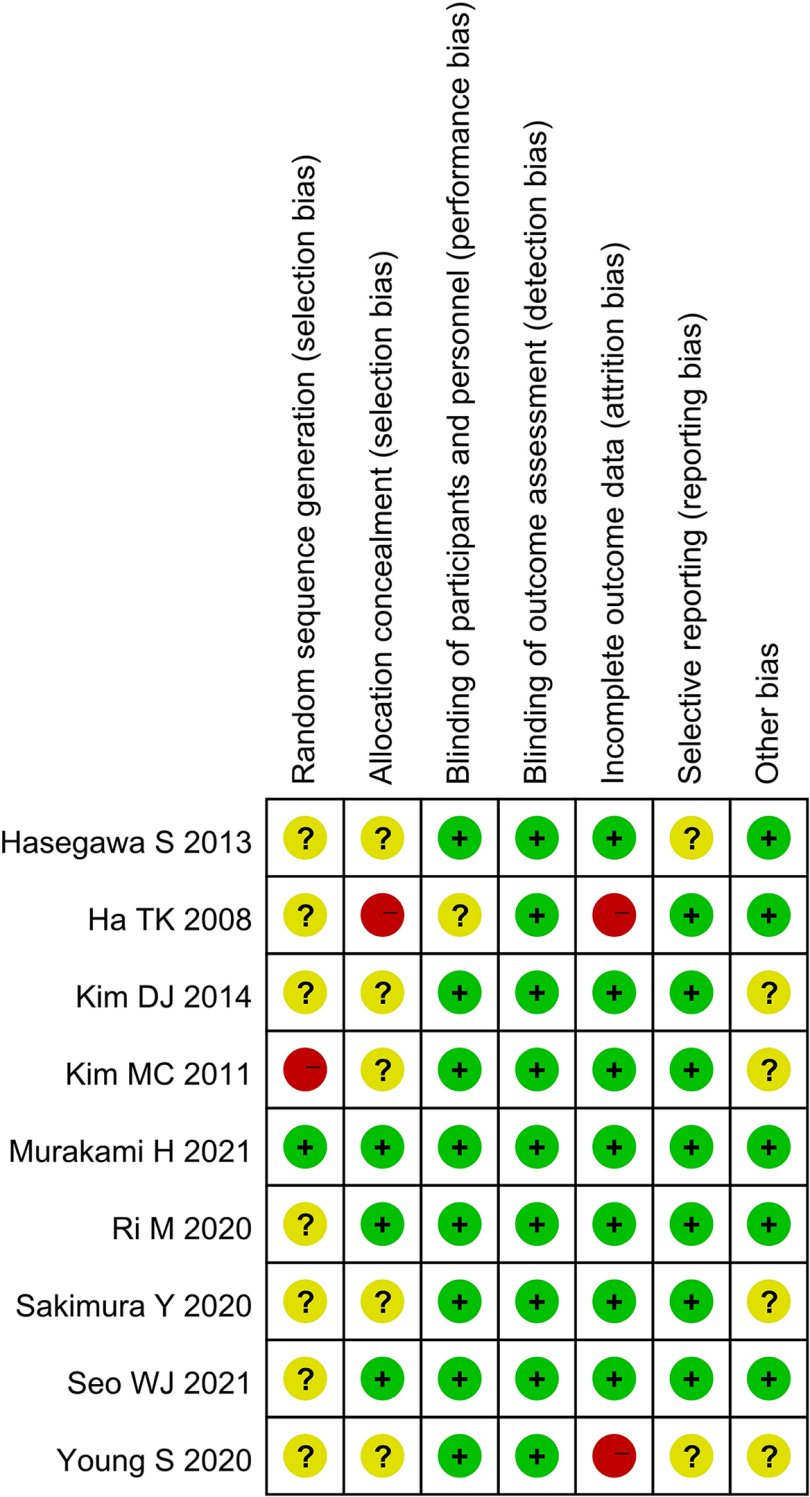
Risk of bias summary for the included studies.

A total of 920 studies were identified. Nine studies (3–5, 7–12), including 8 retrospective studies and 1 randomized controlled trial, were ultimately included in this meta-analysis. Three multicenter studies were obtained. The details of the selection procedures were in line with the PRISMA flowchart (**Figure 1**). General information from the included studies is summarized in **Table 1**. The total number of included patients with GC was 3335 (1372 in the OPG group and 1963 in the GCO group). These studies were from three countries (i.e., Japan, Korea, and USA) and published from 2008 to 2021. The sample size ranged from 37 patients to 1116 patients. Additionally, the open gastrectomy was the most frequently performed operation method in these studies. According to the Cochrane Handbook, nine studies were at slight or moderate risk of bias. The items evaluated for each study are shown in **Figure 2**.

**Table 1:** Characteristics of included studies.

Reference	Published year	Country	Study interval	Study design	Clinical object	Pathological stage	Sample size (OPG : GCO)	Operation method
Hasegawa et al. (3)	2013	Japan	2000-2009	S; R; PSM	AGC	pT2-4N0-3	98:98	OG or LG
Ha et al. (7)	2008	Korea	2004-2006	S; R	EGC	pT1-4	124:992	OG
Kim et al. (8)	2014	Korea	2004-2011	S; R	AGC	pT2-3N0-3	66:80	LG
Kim et al. (9)	2011	Korea	2005-2006	S; R	EGC	pT1-2N0-1	17:20	OG
Murakami et al. (4)	2021	Japan	2011-2018	M; RCT	AGC	pT1-4N0-3	125:122	OG
Ri et al. (10)	2020	Japan	2006-2012	M; R; PSM	AGC	pT1-4N0-3	263:263	OG
Seo et al. (5)	2021	Korea	2003-2015	S; R; PSM	AGC	pT3-4N0-3	225:225	OG or LG
Sakimura et al. (11)	2020	Japan	2008-2017	S; R; PSM	AGC	pT1-4N0-3	73:73	OG or LG
Young et al. (12)	2020	USA	2008-2016	M; R	AGC	pT1-4N0-3	381:90	OG

OPG, omentum-preserving gastrectomy; GCO, gastrectomy with complete omentectomy; S, single centre; M, multicentre; R, retrospective study; RCT, randomized controlled trial; PSM, propensity score matching; EGC, early gastric cancer; AGC, advanced gastric cancer; OG, open gastrectomy; LG, laparoscopic gastrectomy.

### Patient- and Tumor-Related Baseline Characteristics

**Figure 3:**
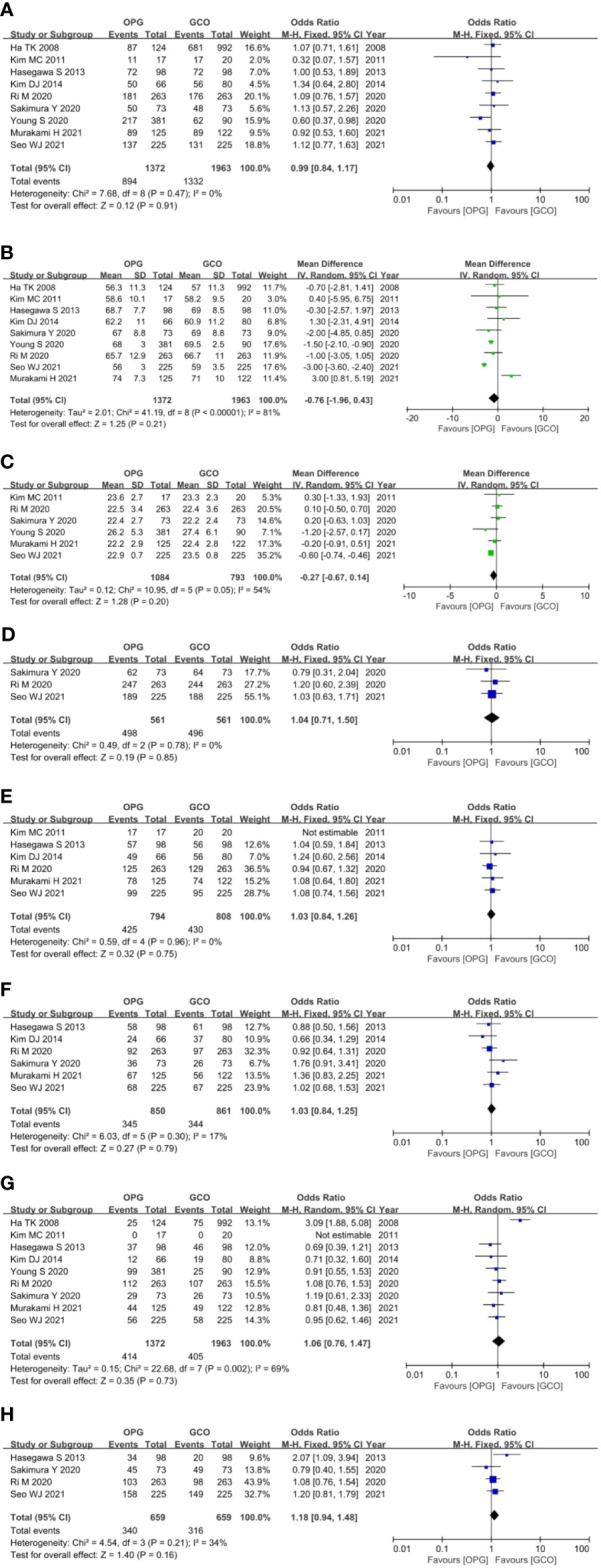
Forest plots showing the assessment of baseline features including **(A)** sex, **(B)** age, **(C)** body mass index, **(D)** American Society of Anesthesiologists score, **(E)** pathological stage, **(F)** histologic type, **(G)** resected type and **(H)** adjuvant chemotherapy. OPG, omentum-preserving gastrectomy; GCO, gastrectomy with complete omentectomy.

For the patient- and tumor-related variables, sex (male and female), age (mean ± SD), BMI (mean ± SD), ASA score (ASA 1/2 and ASA 3/4), pathological stage (stages 1/2 and 3/4), histologic type (differentiated and other types), resected type (total and subtotal gastrectomy), and adjuvant chemotherapy (with and without) were analyzed. All variables of the OPG and GCO groups were comparable and analyzed using the fixed- (*I* ^2^ < 50%) or random-effects model (*I* ^2^ > 50%). As shown in **Figure 3**, the baseline parameters between the two groups were not statistically significant (p > 0.05).

### Surgical Outcomes

**Figure 4:**
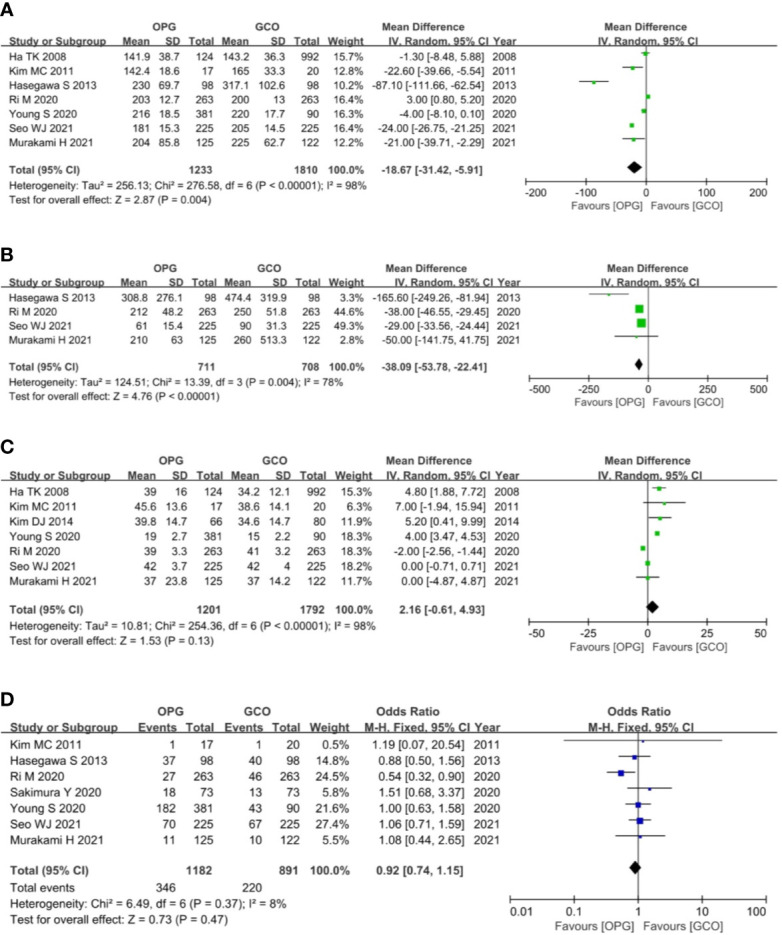
Forest plots showing the assessment of surgical outcomes including **(A)** operative time, **(B)** intraoperative blood loss, **(C)** the number of dissected lymph nodes and **(D)** postoperative complications. OPG, omentum-preserving gastrectomy; GCO, gastrectomy with complete omentectomy.

Seven studies (3–5, 7, 9, 10, 12) reported the operation time of both groups, and the OPG group was associated with shorter operative time (MD = −18.67, 95% CI = −31.42 to −5.91, P = 0.004) than the GCO group (**Figure 4A**). Four studies (3–5, 10) reported the intraoperative blood loss of both groups, and the OPG group was related to less intraoperative blood loss (MD = −38.09, 95% CI = −53.78 to −22.41, P < 0.00001) than the GCO group (**Figure 4B**). Seven studies (4, 5, 7–10, 12) reported the number of retrieved lymph nodes of both groups, and the difference between the two groups was not statistically significant (MD = 2.16, 95% CI = −0.61 to 4.93, P = 0.13) (**Figure 4C**). Seven studies (3–5, 9–12) reported the postoperative complications of both groups, and no significant difference between the OPG and GCO groups was observed (OR = 0.92, 95% CI = 0.74 to 1.15, p = 0.47) (**Figure 4D**).

### Oncological Outcomes

**Figure 5:**
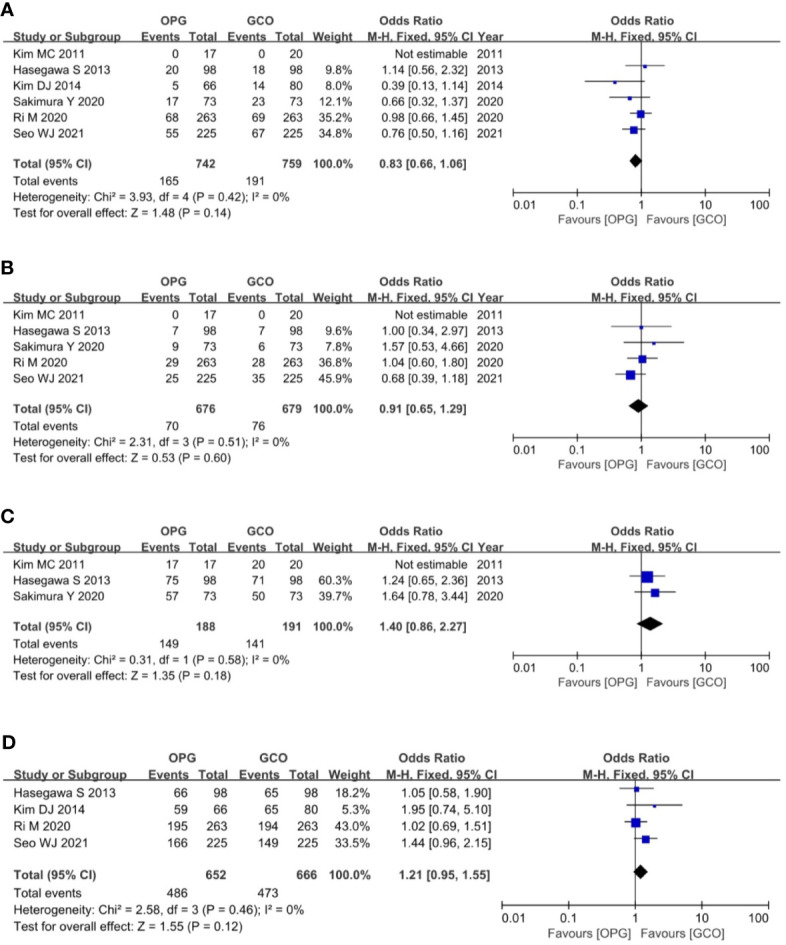
Forest plots showing the assessment of oncological outcomes including **(A)** overall recurrence rate, **(B)** peritoneal recurrence rate, **(C)** 3-year RFS rate and **(D)** 5-year RFS rate. OPG, omentum-preserving gastrectomy; GCO, gastrectomy with complete omentectomy; RFS, relapse-free survival.

Six studies (3, 5, 8–11) reported the overall recurrence rates, and five studies (3, 5, 9–11) reported the peritoneal recurrence rates. These studies expatiated and compared the recurrence rate and type between the two groups. Recurrence patterns were classified as recurrence of primary site, peritoneum, lymph node, liver, lung, bone, and combined metastasis. The meta-analysis of pooled analysis showed no significant difference in the overall (OR = 0.83, 95% CI = 0.66 to 1.06, p = 0.14) (**Figure 5A**) and peritoneal (OR = 0.91, 95% CI = 0.65 to 1.29, p = 0.60) (**Figure 5B**) recurrence rates of OPG and GCO groups.

The primary outcome of this study was the assessment of the RFS rate of OPG in patients with GC. Ultimately, three studies (3, 9, 11) reported the 3-year RFS rates, and the meta-analysis of pooled analysis showed no significant difference in the 3-year RFS rate between OPG and GCO groups (OR = 1.40, 95% CI = 0.86 to 2.27, p = 0.18) (**Figure 5C**). Four studies (3, 5, 8, 10) reported the 5-year RFS rates, and the meta-analysis of pooled analysis showed that the 5-year RFS rates of the two groups were similar (OR = 1.21, 95% CI = 0.95 to 1.55, p = 0.12) (**Figure 5D**).

### Publication Bias

**Figure 6:**
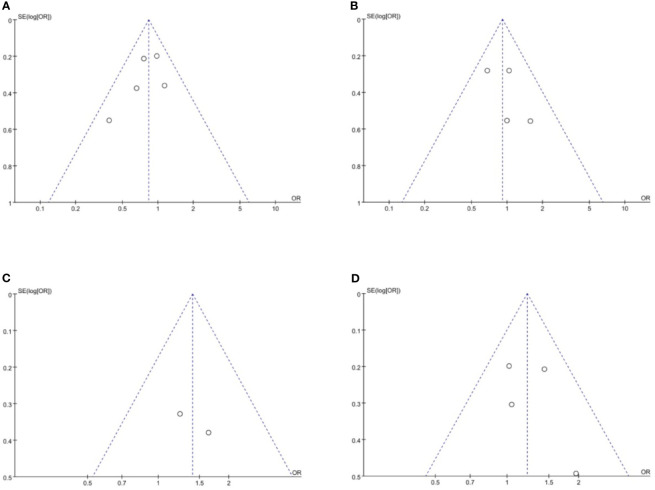
Funnel plots of publication bias based on **(A)** overall recurrence rate, **(B)** peritoneal recurrence rate, **(C)** 3-year RFS rate and **(D)** 5-year RFS rate. RFS, relapse-free survival.

Funnel plots were used to assess the potential publication bias in the meta-analysis. As shown in **Figure 6**, although these funnel plots were symmetrical, we maintain that there were medium risk of publication bias because of insufficient RCT articles.

## Discussion

In a clinical practice, the Japanese Gastric Cancer Treatment Guidelines (5th edition) indicate that removal of the greater omentum is usually recommended in the standard gastrectomy for T3 or deeper tumors (2). Also, the National Comprehensive Cancer Network Guidelines Version 1 mentions that the D1 dissection entails the resection of the greater and lesser omenta (13). Thus, until now, GCO is considered to be essential for GC surgery and is performed worldwide. The greater omentum is usually resected to eliminate the microscopic seeds on the assumption that peritoneal dissemination may be increased by preservation of the greater omentum. But it is impossible for surgeons to completely remove the peritoneum from the abdominal cavity. Furthermore, several retrospective studies showed that OPG does not increase the recurrence rate and worsen the survival of patients with GC compared with GCO (3, 5, 10). Sakimura Y et al. (11) reported that the recurrence rates of OPG and GCO groups are not different and that omentectomy is not required for radical gastrectomy. Seo WJ et al. (5) and Ri M et al. (10) reported no survival difference between OPG and GCO in patients with GC. Based on our meta-analysis, no significant difference is observed in the overall and peritoneal recurrence rates of OPG and GCO groups (p > 0.05). However, there is a trend that OPG is related to lower overall recurrence rate, which was 22.2% and that was 25.2% in GCO group. Generally, OPG could cause peritoneal recurrence, but the peritoneal recurrence rates were basically comparable, which were 10.4% and 11.2% for OPG and GCO groups, respectively. So the reason for this trend maybe the fact that there were less patients completed adjuvant chemotherapy in GCO group, the rates of which were 51.6% and 47.9% for OPG and GCO groups, respectively. The 3- and 5-year RFS rates between the two groups are comparable (p > 0.05). These results indicate that OPG may be an oncologically safe procedure for patients with GC.

The greater omentum is an important intra-abdominal organ and occupies a central position in peritoneal defense mechanisms. It achieves this through its innate immune function, high absorptive capacity, and ability to adhere to adjacent structures to seal off gastrointestinal defects and promote their healing with its pronounced angiogenic activity (14, 15). Additionally, GCO may cause abdominal complications, such as injury to spleen, colon, or mesocolon. Therefore, in managing patients with intra-abdominal malignancies, omentectomy requires further study to determine its association with a clear survival advantage and evaluate how much needs to be removed. Murakami H et al. (4) pointed out that OPG can reduce operation time and intraoperative blood loss. Indeed, performing GCO in abdominal operation especially in laparoscopic gastrectomy is technically difficult and time-consuming. Recently, several clinical trials of laparoscopic gastrectomy for GC are ongoing, and the laparoscopic gastrectomy for GC has become widespread worldwide (16–18). OPG may shorten the operation time and is helpful for surgeons to carry out laparoscopic surgery technically. According to our analysis, OPG is associated with shorter operative time (P = 0.004) and less intraoperative blood loss (P < 0.00001) than GCO, but the number of dissected lymph nodes (P = 0.13) and postoperative complications (P = 0.47) of the two groups are comparable. These results suggest that OPG is beneficial for surgical outcomes for patients with GC.

Our study indicates two important findings. First, OPG does not affect the overall and peritoneal recurrence rates and the 3- and 5-year RFS rates of patients with GC. Second, OPG can reduce operative time and intraoperative blood loss but cannot reduce the number of retrieved lymph nodes and increase postoperative complications. These results support our hypothesis that omentectomy can be omitted during GC surgery in terms of short- and long-term outcomes. However, controversy about the contribution of surgical intervention to the elimination of cancer cells for the prevention of peritoneal relapse still exists (19–21). Jongerius EJ et al. (20) pointed out that the incidence of metastases in the greater omentum is low in resectable GC and is associated with advanced disease and nonradical features. Thus, omentectomy, as part of a radical gastrectomy, may be omitted. Exactly, a large-scale randomized controlled trial indicates that the micrometastatic disease in patients who received curative surgery for GC can be eliminated by systemic chemotherapy rather than surgical intervention (22). Expectantly, a large-scale randomized controlled trial about assessment of OPG for patients with GC in Japan (JCOG1711, UMIN000036253) (23) is ongoing, and the result of this study may confirm OPG as the new standard in the future.

## Conclusions

Despite the limitations of the included studies, this meta-analysis indicates that OPG might be an oncologically safe procedure with better surgical outcomes for patients with GC than GCO. Nevertheless, high-quality prospective studies and randomized controlled trials are needed to confirm this benefit.

## Data Availability Statement

The original contributions presented in the study are included in the article/supplementary material. Further inquiries can be directed to the corresponding author.

## Author Contributions

ZL and MS made substantial contributions to conception and design for this work. ZL, MS, and YZ collected all the data. ZL and MS was the major contributor in writing the manuscript. HJ, LX, ZH, YL, YJ and XL performed critical revision for important intellectual content. All authors contributed to the article and approved the submitted version.

## Funding

This work was supported by (1) National Natural Science Foundation of China (No. 30672058); (2) Scientific Research Project of Southwest Medical University (No. 2020ZRQNB026).

## Conflict of Interest

The authors declare that the research was conducted in the absence of any commercial or financial relationships that could be construed as a potential conflict of interest.

## Publisher’s Note

All claims expressed in this article are solely those of the authors and do not necessarily represent those of their affiliated organizations, or those of the publisher, the editors and the reviewers. Any product that may be evaluated in this article, or claim that may be made by its manufacturer, is not guaranteed or endorsed by the publisher.
